# Unique pioneer microbial communities exposed to volcanic sulfur dioxide

**DOI:** 10.1038/srep19687

**Published:** 2016-01-21

**Authors:** Reiko Fujimura, Seok-Won Kim, Yoshinori Sato, Kenshiro Oshima, Masahira Hattori, Takashi Kamijo, Hiroyuki Ohta

**Affiliations:** 1 Ibaraki University College of Agriculture, Ibaraki 300-0332, Japan; 2 Department of Computational Biology, Graduate School of Frontier Science, The University of Tokyo, Kashiwa, Chiba 277-8568, Japan; 3 National Research Institute for Cultural Properties, Tokyo, Tokyo, 110-8713, Japan; 4 Graduate School of Life and Environmental Science, University of Tsukuba, Tsukuba, Ibaraki 305-8572, Japan

## Abstract

Newly exposed volcanic substrates contain negligible amounts of organic materials. Heterotrophic organisms in newly formed ecosystems require bioavailable carbon and nitrogen that are provided from CO_2_ and N_2_ fixation by pioneer microbes. However, the knowledge of initial ecosystem developmental mechanisms, especially the association between microbial succession and environmental change, is still limited. This study reports the unique process of microbial succession in fresh basaltic ash, which was affected by long-term exposure to volcanic sulfur dioxide (SO_2_). Here we compared the microbial ecosystems among deposits affected by SO_2_ exposure at different levels. The results of metagenomic analysis suggested the importance of autotrophic iron-oxidizing bacteria, particularly those involved in CO_2_ and N_2_ fixation, in the heavily SO_2_ affected site. Changes in the chemical properties of the deposits after the decline of the SO_2_ impact led to an apparent decrease in the iron-oxidizer abundance and a possible shift in the microbial community structure. Furthermore, the community structure of the deposits that had experienced lower SO_2_ gas levels showed higher similarity with that of the control forest soil. Our results implied that the effect of SO_2_ exposure exerted a selective pressure on the pioneer community structure by changing the surrounding environment of the microbes.

Volcanic eruptions offer numerous opportunities to increase our understanding of terrestrial ecosystem formation. The first key step of the formation process is the input of biologically transformed atmospheric carbon and nitrogen to the ecosystem by pioneer microorganisms^1^,^2^,^3^. Most of the early microbial colonizers probably have CO_2_ and N_2_ fixation capabilities, and they can grow by utilising photo- or chemolithotrophic energy-generation systems (i.e., by oxidation of inorganic substrates: e.g., S^0^, Fe^2+^, CO and H_2_). As a result, their activity promotes the growth of diverse heterotrophic communities, which are associated with the physicochemical changes of surrounding microenvironments^4^,^5^. However, knowledge of the development process of their ecosystems and the mechanisms in the initial stage of the formation is still limited. This study investigated the succession of the early microbial communities in fresh volcanic deposits (<10 years old) which were exposed to volcanic sulfur dioxide (SO_2_) over long periods. The aim was to develop an understanding of the association between microbial succession and changing environmental conditions.

The island of Miyake (our study site, Miyake-jima; Fig. 1a,b) released a large amount of volcanic SO_2_ gas from the newly formed crater after the eruption of Mt. Oyama in 2000. The daily average rates of SO_2_ gas emission peaked at 54 kt d^−1^ in December 2000 and decreased to 7 kt d^−1^ by the end of 2002^6^. Although the amount of SO_2_ emission had further decreased over the past ten years, it was still detectable in 2014 (see details in the Materials section and Supplementary Table S1). The volcanic activity destroyed plant ecosystems through SO_2_ exposure and volcanic ash deposition and buried mature soil ecosystems^7^. Likewise, the newly deposited volcanic ash was acidified by exposure to SO_2_ gas^8^.

Previously, we reported that nitrogen-fixing obligate autotrophic iron-oxidizing bacteria were the dominant group in the bacterial community structure in young deposits of volcanic ash from Miyake-jima (<6.6 years old)^9^. However, the early colonization of iron oxidizers was not found to be uniform among newly formed substrates in other terrestrial volcanoes. For example, Hawaiian lava and Mt. Pinatubo lahar deposits showed the importance of different types of chemolithotrophs as pioneer organisms (i.e., CO- and H_2_-oxidizing bacteria) which utilise atmospheric trace gas as energy substrates^3^,^10^,^11^. Additionally, a study of the Fimmvörðuháls lava flow of the Eyjafjallajökull volcano on Iceland reported the dominance of *Betaproteobacteria* related to non-phototrophic diazotrophs and chemolithotrophs such as *Herbaspirillum* and *Thiobacillus*, respectively^12^. Thus, the dominance of acidophilic iron-oxidizers as pioneer organisms is a unique characteristic of fresh volcanic deposits from Miyake-jima.

The goal of this study was to gain deeper insight into the succession of structure and function of pioneer microbial communities on volcanic ash deposits from Miyake-jima. The present study adopted metagenomic analysis of the microbial ecosystem to examine in-depth entire environmental genomes and thus to obtain more succession results with reduced bias and to incorporate additional data such as microbial functional information.

## Results and Discussion

### Chemical characteristics of the fresh volcanic ash deposits

**Figure 1:**
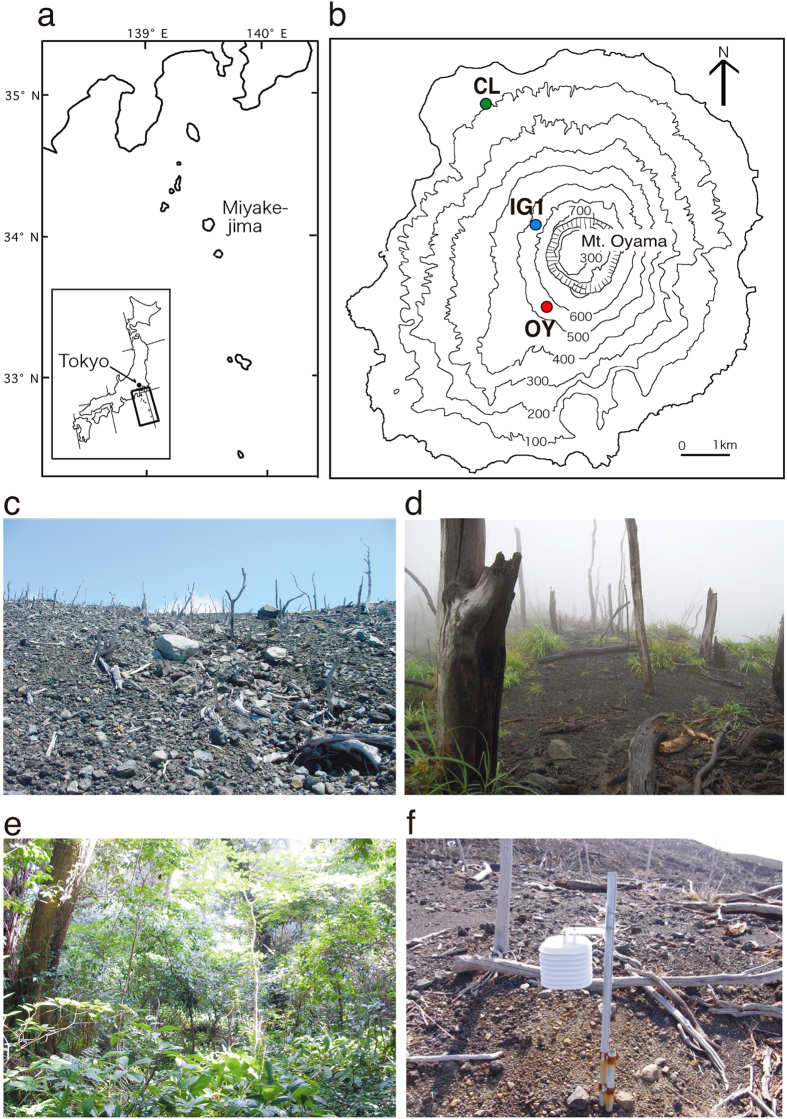
Map of the study site, locations and photographs of sampling sites. (**a**) Map showing the location of Miyake-jima on the western rim of the Pacific Ocean. (**b**) Three study sites on the island. Colour plots show each site (red, OY; blue, IG1; green, CL). (**c**–**e**) Photographs of each site (**c**, site OY in 2005; **d**, site IG1 in 2009; **e**, site CL in 2005). (**f**) SO_2_ data logger. The map was created using Adobe Illustlater CS6.

We analysed the volcanic ash deposits derived from a site heavily affected by SO_2_ exposure (site OY; Fig. 1b,c). Time-series samples were taken 3.5, 6.6 and 9.5 years after the eruption (3.5-, 6.6- and 9.5-OY volcanic deposit [VD], respectively). Those datasets were compared with an 8.5-year-old deposit derived from a less-affected site (site IG1; IGVD; Fig. 1b,d), and with forest soil derived from an undamaged site on the island (control sample)^13^,^14^ (site CL; CLS; Fig. 1b,e). The impact of long-term SO_2_ exposure had decelerated the recovery of vegetation and had acidified the deposits^7^,^8^. This effect was heavier on the leeward side than on the windward side of Mt. Oyama (OY and IG1 sides, respectively) because the monsoon wind over the island (i.e., westerly and north-easterly winds) affected the direction of the volcanic gas flow^7^,^15^. Reference for the volcanic exposure frequency at each site, SO_2_ gas monitoring data suggested that the detection frequency of atmospheric SO_2_ gas was the highest in site OY (Supplementary Table S2).

**Figure 2:**
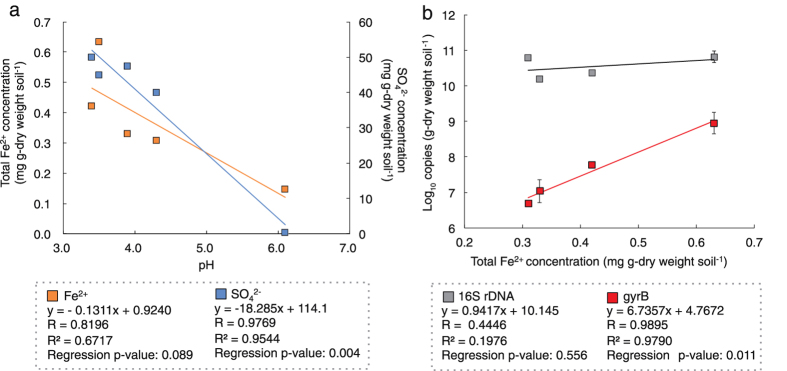
Relationships among chemical compositions and bacterial numbers. (**a**) Relationship between pH and SO_4_^2−^ (blue) or Fe^2+^ (orange) concentration and (**b**) Fe^2+^ concentration and copy number of bacterial 16S rRNA gene (grey) or *L. ferrooxidans gyrB* gene (red). Error bars, s.d. of triplicate results. Result of the statistical significance test (R, correlation coefficient; R^2^, determination coefficient of the regression model).

Time-series samples on site OY (OYVDs) were characterised by lower pH (3.4–3.9), as well as by total carbon (TC) content and nitrogen (TN) content (≤0.5 and ≤0.1 g kg^−1^, respectively) higher than those on sites IGVD and CLS (Table 1). The Pearson’s correlation coefficient (R) for each dataset of chemical properties suggested high correlations between ferrous ion (Fe^2+^) concentration and pH, as well as SO_4_^2−^ concentration and pH (Fig. 2a). The concentration of Fe^2+^, which could be considered a critical factor affecting the abundance of iron oxidizers, was negatively correlated with the pH of the deposit (R = 0.8196, p = 0.089); The instability of ferrous iron above pH 4 supported this finding^16^,^17^,^18^. The clear inverse correlation between SO_4_^2−^ concentration and pH (R = 0.9769, p = 0.004) suggested the acidification of the deposits caused by SO_4_^2−^ accumulation due to SO_2_ exposure, as well as previous reports^8^,^19^. Therefore, these relationships suggest that the chemical properties of the OYVD deposits had been affected by SO_2_ gas exposure.

### Microbial community succession on the fresh volcanic ash deposits

**Figure 3:**
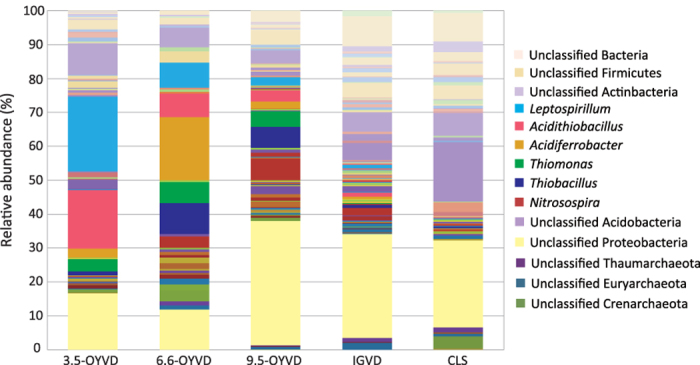
Structure of the prokaryotic microbial community at the genus level. The relative abundance of each genus was calculated as the ratio of individual 16S rRNA gene assigned sequences to the total number of sequences. Colour legends of major genera are shown; those of minor genera are shown as original data in Supplementary Table S4.

**Figure 4:**
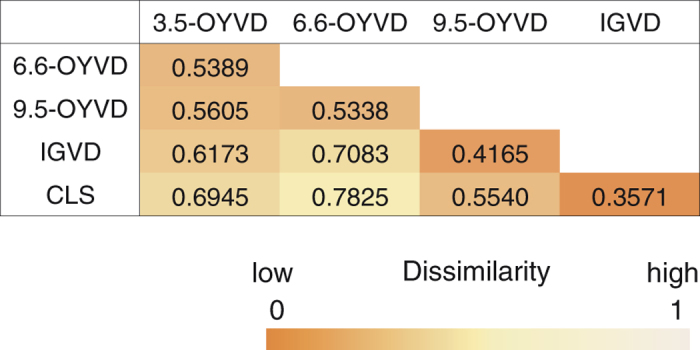
Colour-mapped matrix table of Bray−Curtis dissimilarity indices. Indices were calculated by using microbial abundance patterns of genus-assigned reads. The dissimilarity indices indicate that the lower number is more similar (i.e., not dissimilar) between samples. The colour indicator is shown in the figure.

Prokaryotic 16S rRNA gene (16S rDNA) sequences were obtained from the metagenomic datasets (Supplementary Table S3). The microbial community structure indicates that the most abundant genus was *Leptospirillum*, which accounted for 22% of the total 16S rDNA gene sequences in 3.5-OYVD (Fig. 3 and Supplementary Table S4). However, this ratio decreased in 6.6-OYVD (14%). A similar tendency to that of *Leptospirillum* was observed in the second most abundant genus, *Acidithiobacillus*; the percentages of this group were 17 and 7% in 3.5- and 6.6-OYVDs, respectively. Moreover, we found that *Leptospirillum ferrooxidans* and *Acidithiobacillus ferrooxidans*, obligate autotrophic acidophilic nitrogen-fixing bacteria which can utilize ferrous iron as an energy source, were the most abundant species in those genera in the fresh volcanic ash deposits (data not shown). These results were consistent with Fujimura *et al.* (2012), except for the non-decreasing relative abundance of *Acidithiobacillus* between 3.5- and 6.6-OYVDs (16 and 20%, respectively). Incidentally, this study revealed diverse communities more than that in our previous report (PCR-clone library analysis), because community analysis using metagenomic dataset enabled detection of many small groups by obtaining a large number of sequences (average: 470) with less bias. Meanwhile, the relative abundances of *Leptospirillum* and *Acidithiobacillus* in 9.5-OYVD (3 and 4%, resp.) and IGVD deposits were low (0 and 1%, resp.; Fig. 3 and Supplementary Table S4). Interestingly, IGVD community seems to have the mosaic structure of the 9.5-OYVD and CLS communities; but the dissimilarity index (Fig. 4, discussed below) indicated that the IGVD community was more similar to the CLS community than to the 9.5-OYVD community (Fig. 4 and Supplementary Fig. S1). These results suggested that the initial stage of the microbial community could easily change because of age or changes in the environment. Furthermore, the community structure of CLS showed a high abundance of *Acidobacteria* and *Actinobacteria* groups, but *Acidithiobacillus* and *Leptospirillum* were undetectable (Fig. 3 and Supplementary Table S4). The CLS community was similar with our previous study in 2005^9^ (CLS of this study was taken in 2007), and it suggests that the community structure of the forest soil is more stable than is the volcanic ash community.

*Leptospirillum* and *Acidithiobacillus* are commonly dominant in highly acidic environments such as acid mine drainages^16^,^20^,^21^, because such environments are rich in their energy substrates (e.g., sulfide and Fe^2+^)^22^. The abundance of these organisms in the OYVDs was also probably affected by environmental conditions such as Fe^2+^ concentration and acidity; therefore, we compared the relationship between the population size of *Leptospirillum* (a strict iron oxidizer) and the chemical properties of the deposits (Table 1). Quantitative PCR analysis of the *L. ferrooxidans* gene encoding for DNA gyrase subunit B (*gyrB*) showed different population sizes among the volcanic ash deposits. The highest correlation was obtained with the Fe^2+^ concentration (Fig. 1b; R = 0.990, p = 0.01), and a negative correlation was obtained with pH and SO_4_^2−^ concentration (R = 0.797 and 0.308, p = 0.20 and 0.69, respectively). Meanwhile, the quantity of 16S rDNA showed very weak correlation with Fe^2+^ concentration (Fig. 1b; R = 0.445, p = 0.56), but it was correlated with SO_4_^2−^ concentration (negative correlation; R = 0.752; p = 0.248). These results imply that the concentrations of inorganic energy substrates for the dominant groups could be a key environmental factor for the microbial community succession in the fresh volcanic ash deposit.

Archaeal contribution to the early ecosystem development in fresh volcanic ash deposits from Miyake-jima seems to be very small because their 16S rDNA sequences in 3.5- and 6.6-OYVDs were undetectable in the community analysis (Fig. 3 and Supplementary Table S4). This result confirms that the PCR assay of the archaeal 16S rDNA amplification products were undetectable despite the use of some archaeal universal primer sets (data not shown). The archaeal 16S rDNA sequence reads were detected in 9.5-OYVD, IGVD and CLS, which respectively accounted for 1.3, 3.6 and 6.5% of the total 16S rDNA (Supplementary Table S4).

Photosynthetic microbes were also not detected in the volcanic ash deposits (Fig. 3 and Supplementary Table S4). Cyanobacterial photosynthesis is one of the important activities in the formation of initial soil ecosystems, which contribute to carbon and nitrogen accumulation, and soil aggregate formation in such ecosystems^2^,^5^. However, previous reports indicate that sulfite inhibits photosynthetic activity^23^,^24^,^25^; therefore, SO_4_^2−^ accumulation due to SO_2_ exposure probably repressed the colonization of the photosynthetic microbes, and SO_4_^2−^ may therefore be involved in the selection of pioneer communities in the OYVDs.

Bray−Curtis dissimilarities calculated from the relative abundance of each assigned genus (Fig. 4) indicate the similarity of the community composition patterns among samples. The results show the highest similarity between IGVD and CLS and the lowest similarity between 6.6-OYVD and CLS (Fig. 4 and Supplementary Fig. S1; dissimilarity values of 0.36 and 0.78, respectively). Interestingly, the IGVD community was more similar to CLS than to 9.5-OYVD despite the deposit being younger than 9.5-OYVD. If the CLS community was a mature ecosystem, then the IGVD community would had been more developed than the oldest OYVD community. This result suggests that SO_2_ exposure leads to differences in the community composition by changing the environmental conditions.

### Carbon- and nitrogen-fixing microbial communities and their succession

**Figure 5:**
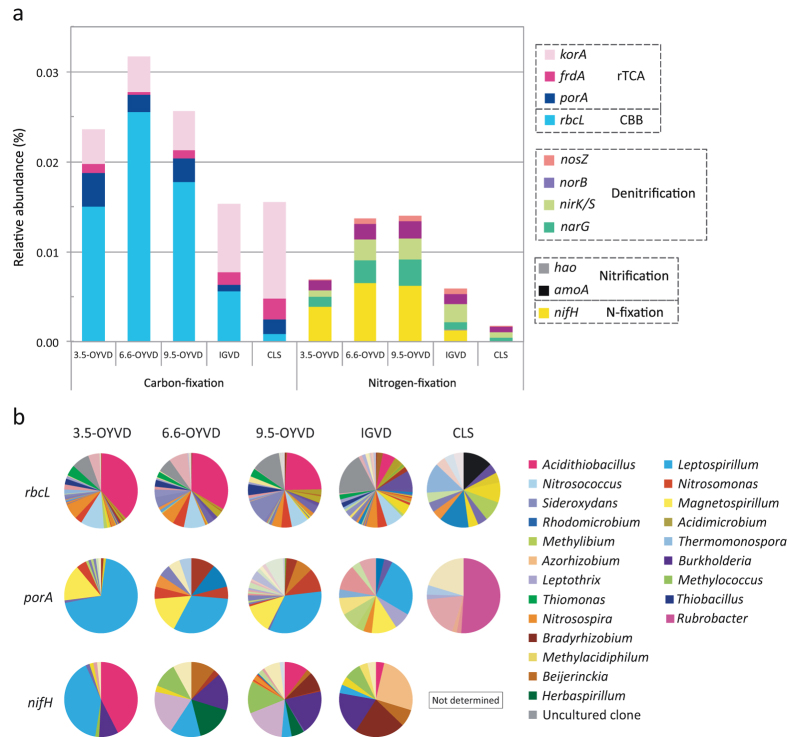
Relative abundances of carbon-fixation and nitrogen-cycling genes and taxonomic assignments of carbon and nitrogen fixers. (**a**) Relative abundances of targeted genes were calculated as the ratio of predicted ORFs in each sample to the total number of ORFs (Supplementary Table S3). Colour legends are shown. (**b**) Relative abundances of the taxonomic assignments of the *rbcL*, *porA* and *nifH* genes were calculated as the ratio of each assigned gene sequence to the total number of sequences. Colour legends of major genera are shown in this figure; those of minor genera are shown as original data in Supplementary Table S5.

Microbial communities with the selected genes responsible for CO_2_ fixation were analysed. These genes are related to the Calvin–Benson–Bassham cycle (CBB; *rbcL*) and the reductive tricarboxylic acid cycle (rTCA; *korA*, *frdA* and *porA*). The relative abundances of these genes, particularly that of the *rbcL* gene, were higher in OYVDs than in IGVD and CLS (Fig. 5a). Taxonomic assignments of *rbcL* genes showed the predominance of *Acidithiobacillus* in OYVDs (24–38% of total *rbcL* genes; Fig. 5b and Supplementary Table S5). Another abundant iron-oxidizing bacterium, *Leptospirillum*, lacks the CBB system but possesses the rTCA system. The *porA* gene sequences derived from *Leptospirillum* accounted for 70% of the total *porA* genes in 3.5-OYVD and 30% of genes in 6.6- and 9.5-OYVDs (Fig. 5b). Archaeal *rbcL* and *porA* gene abundances showed the highest total percentages in IGVD and 9.5-OYVD, respectively (Supplementary Table S5). Furthermore, cyanobacterial CO_2_ fixation genes were undetectable in OYVDs but were present in IGVD and CLS.

The abundance of the nitrogen fixation gene (*nifH*) was one of the striking features of OYVDs (Fig. 5a), accounting for ~50% of nitrogen-cycling-related genes. A comparison of the percentage of *nifH* and the combined abundance of denitrification-related genes (*narG, nirK/S, norB* and *nosZ*) showed the relatively low abundance of *nifH* in IGVD and its undetectable concentrations in CLS. The latter result might had been caused by the relatively low coverage and the high diversity of genes in the CLS metagenome. Taxonomic assignment of the *nifH* gene indicates that while both *Acidithiobacillus* and *Leptospirillum* accounted for as much as 80% of the sequences in 3.5-OYVD, they accounted for lower percentages in 6.6- and 9.5-OYVDs (Fig. 5b and Supplementary Table S5). In these sites, the composition of microbes shifted from an obligate chemolithotrophic community to heterotrophic and/or facultative lithotrophic diazotrophs such as *Burkholderia*, *Beijerinckia* and *Leptothlix*. These results thus supported the significance of iron-oxidizing bacteria in the early ecosystem and confirm that the process of succession differs between nitrogen- and carbon-fixing microbial communities.

The box-plot of Bray−Curtis dissimilarity indices showed high similarity of functional gene compositions among samples (indices <0.5); meanwhile, the community compositions among samples were more diverse (Supplementary Fig. S2). This result implies that the essential housekeeping genes for the maintenance of basic cellular functions in most of the microbes were universally abundant in each metagenome^26^,^27^. However, non-housekeeping genes such as those for degradation of organic contaminants were also detectable in OYVDs and IGVD, occurring at ratios similar to those in CLS (data not shown). This detection suggest that most of the potential function, which is the same as for the forest soil, is already present in the early microbial ecosystem even with the lack of a taxonomically diverse community. Nonetheless, the relative abundance of distinguishing non-housekeeping genes (e.g., *rbcL*) could probably be affected by the abundance of each distinguishing taxonomic group.

Our metagenomic analysis illuminates the process of succession in early microbial ecosystems in acidic fresh volcanic ash deposits, despite the limitations inherent to our study, such as the statistical significance due to the limited number of samples. We observed a unique pioneer community and its succession in less than 10 years on volcanic ash deposits at a fixed site, which seems to reflect the peculiar environment of Miyake-jima. One possible reason for the short-term microbial succession is that environmental factors easily affected the community on the deposit because the volcanic ash deposit had not formed complex structures such as soil aggregates (i.e., smaller buffering effect and less-complex habitats than those of matured soil). Therefore, the change in the volcanic SO_2_ effect had an important impact on the initial stage of ecosystem formation process in Miyake-jima.

## Materials and Methods

### Materials

Miyake-jima is a basaltic volcanic island located on the Izu-Mariana arctic ridge in the Pacific Ocean, approximately 180 km south of Tokyo (34°05′ N, 139°31′E; Fig. 1a). Mt. Oyama, the active volcano situated in the centre of the island, last erupted in 2000. This eruption ejected a large amount of volcanic SO_2_ gas from a newly created summit caldera, contributing a significant amount to the global rate of SO_2_ gas emission from non-erupting volcanoes (estimated at 26 kt d^−1^)^6^. The daily emission of SO_2_ gas was ~48,000 t d^−1^ immediately after the eruption^19^; it then declined slowly after the eruption (Supplementary Table S1). Approximately 283 t d^−1^ was still being emitted in 2014 (Japan Meteorological Agency, http://www.data.jma.go.jp/svd/vois/data/tokyo/320_Miyakejima/320_So2emission.htm). The SO_2_ gas had a massive impact on the island ecosystem, destroying ~60% of island vegetation and affecting vegetation recovery^7^. In addition, the volcanic ash deposits were acidified as a result of SO_4_^2−^ absorption due to SO_2_ gas exposure^8^. Kato *et al.* (2002)^8^ reported that the fresh volcanic ash showed high content of fine sand (36–76%), acidity [pH (H_2_O) 3.1–4.0], and high amounts of exchangeable Ca^2+^ (33.5–115 cmolc kg^−1^) and Al^3+^ (0.8–10.2 cmolc kg^−1^).

The volcanic ash deposits of the 2000 eruption were taken from an unvegetated site, OY (34°04.69′N, 139°31.04′E; 553 m a.s.l.; Fig. 1b), on February 2004 (deposit age, 3.5 years; 3.5-OYVD), March 2007 (6.6 years; 6.6-OYVD), and February 2010 (9.5 years; 9.5-OYVD), as described previously^9^. Site OY had no vegetation because of exposure to volcanic gas (Fig. 1c). Another study site, IG1 (34°05.37′N, 139°30.84′E; 547 m a.s.l.; Fig. 1b), was less damaged by volcanic gas exposure than was OY. The gas exposure frequency was lower than that of OY (Supplementary Table S2) because of the position of IG1, which is mostly on the windward side of the island^7^,^8^. Site IG1 supported a patchy vegetation of a pioneer grass, *Miscanthus condensatus*, and its deposit had higher pH and lower SO_4_^2−^ concentrations than those of the OY deposit (Fig. 1d and Table 1). The volcanic deposits were taken from an unvegetated spot in July 2009 (deposit age, 8.9 years). The forest soil, which had been undisturbed for over 800 years^7^,^13^, was sampled from a site at the foot of the mountain in March 2007 (site CL; 34°06.68′N, 139°30.06′E; 97 m a.s.l.; Fig. 1b). The vegetation of site CL was a climax forest^13^,^14^ (Fig. 1e). The chemical properties of the soil sample are described in Table 1. Each year, volcanic deposits (approx. 1 kg) were sampled at depths between 0.5 and 50 cm from the surface at almost the same point at sites OY and IG1 each. The deposits were mixed in sterile plastic bags and immediately stored at on ice. The number of sampling points was limited because we needed to consider the direction of SO_2_ gas flow, and because we had limited time for sampling. Samples were divided into two portions and kept at 4 °C and at −20 °C until bacteriological analysis and DNA extraction, respectively.

### Volcanic SO_2_ gas monitoring

Volcanic SO_2_ gas concentrations, humidity and temperature were monitored and recorded from December 2011 to April 2012 using a Gasman monitor (Crowcon Detection Instruments Ltd, Abingdon, UK) and a Hobo data logger (Onset Computer Corporation, Cape Cod, MA, USA), respectively (Fig. 1f). Recorded datasets were collected from the fields on December 2011, February 2012 and April 2012. Equipments were installed at 34°04.69′N, 139°30.96′E (site OY), 34°05.37′N, 139°30.83′E (site IG1), and 34°06.71′N, 139°30.08′E (site CL). Atmospheric SO_2_ concentration was measured for one minute every hour. We then converted the dataset to counts data for SO_2_ detection in each detection range, i.e., 1–2, 2–3, 3–4, 4–5 and ≥5 ppm. However, the total number of recording counts varied (including 0 ppm; Supplementary Table S2) because of gas monitor failure in OY and IG1 due to exposure to high concentrations of SO_2_ gas. SO_2_ detection counts data at each concentration were calculated as a percentage of the total number of SO_2_ detection counts.

### Chemical analyses

Measurements of pH, TC content and TN content were performed as described previously^9^. Total ferrous ion concentration was determined by the 2,2′-dipyridyl method using 1 M sodium acetate trihydrate solution (pH 4.8)^28^,^29^. Sulfate was extracted with ultrapure water at a sample to water ratio of 1:2.5 (by mass) in a reciprocal shaker for 1 h at 250 rpm at room temperature. The slurry was centrifuged at 10,000 × g for 3 min, and the supernatant was filtered through a cellulose filter paper (No. 5B; Advantec, Tokyo, Japan) and then through a cellulose ester membrane (0.45 μm; Advantec). The concentration of SO_4_^2−^ was determined by the turbidimetric method based on the reaction of sulphate with barium chloride, resulting in the precipitation of barium sulphate^30^.

### Extraction of DNA of the microbial community

Before DNA extraction, duplicate samples containing 5 g of the deposit (a total of 10 g deposit was used for the extraction) of 3.5- 6.6- and 9.5-OYVD and IGVD were washed with 800 mL of sterilised ultrapure water to prevent undesired precipitation after ethanol treatment during DNA extraction. Total genomic DNA was extracted from samples according to the protocol of Zhou *et al.* (1996)^31^, which was based on lysis using a high-salt extraction buffer (1.5 M NaCl, pH 8.0) and extended heating (65 °C for 2–3 h) of the sample suspension in the presence of sodium dodecyl sulphate, hexadecyltrimethyl ammonium bromide, and proteinase K. Total genomic DNA of CLS (5 g fresh weight, in duplicate) was extracted by using an ISOIL bead-beating kit (Nippon Gene Co. Ltd., Tokyo, Japan) according to the manufacturer’s instructions. We used different extraction methods for CLS because the former method cannot remove humic substances from the solution. Double stranded DNA was quantified by using a Quant-iT PicoGreen assay kit (Invitrogen Life Technologies, Carlsbad, CA, USA).

### Quantitative real-time PCR (q-PCR)

The absolute quantification of bacterial 16S rDNA and the *L. ferrooxidans gyrB* gene encoding the B subunit of DNA gyrase were analysed through the standard curve method of the qPCR assay. The primer sets used for each gene were Qbac-10F (5′-CAGTTTGATCCTGGCTCAG-3′)^9^ and bac-907R (5′-CCGTCAATTCCTTTRAGTTT-3′)^32^ for the 16S rDNA, and Qlfe-42F (5′-CATCAGGGTTCTGGAGGGTC-3′) and lfe-574R (5′-GGGCAAGAGTGTCAAAAAGG-3′) for the *L. ferrooxidans gyrB* gene. The Qlfe-42F/lfe-574R primer set was designed in this study on the basis of the complete genome sequence of *L. ferrooxidans* strain C2-3^33^ by using Primer3Plus software^34^. The specificity of this primer set was confirmed by in silico PCR assay (Primer-BLAST, National Center for Biotechnology Information (NCBI)) and PCR-clone library analysis^9^ of the *gyrB* gene amplification product using the DNA sample of 3.5-OYVD (data not shown). Each forward primer was labelled at the 5′-end with quenching fluorescence dye (Qprobe; J-bio21, Tsukuba, Japan). Details of the Qprobe method and the reaction mixture have been described by Nishizawa *et al.* (2008)^35^. Triplicate q-PCR assays for the *gyrB* gene were performed with an iCycler PCR (Bio-Rad) under the following conditions: 2 min at 95 °C followed by 45 cycles at 95 °C (30 sec), 54 °C (45 sec) and 72 °C (1.5 min) for 16S rDNA; and 2 min at 95 °C followed by 45 cycles at 95 °C (30 sec), 56 °C (45 sec) and 72 °C (1.5 min). The control DNA samples for 16S rDNA and *gyrB* genes, which were used to generate the standard curves, were 0.1 μg of genomic DNA of *Escherichia coli* str. K-12 substr. MG1655 and *L. ferrooxidans* str. C2-3, respectively. Both DNA samples were used in ten-fold dilution series from 10^4^ to 10. The regression of these standard samples showed a high determination coefficient (R^2^ = 0.996 for 16S; 0.999 for *gyrB*). Multiple negative controls (nuclease-free water) were also tested in each run to confirm the non-existence of pseudo-positive products. The means of the triplicate data set were used in Fig. 2b.

### Sequencing, sequence annotation and functional assignment for metagenomic analysis

Approximately 5 μg of each DNA aliquot was sequenced on a Roche 454 GS FLX Titanium system. Artificially redundant sequences were removed by using a 454 replicate filter.

In the extraction of prokaryotic 16S rDNA sequences for prokaryotic community analysis, we constructed an in-house 16S rDNA database by using the NCBI complete and draft genome sequences for 16S rDNA sorting. The bacterial and archaeal 16S rDNA sequences were assigned using the in-house database by BLASTN programme^36^,^37^. The assigned reads were extracted with threshold values which were set to an E value of ≤10^−5^ and a minimum aligned length of ≥100 bp. Extracted 16S rDNA sequences were annotated by BLASTN by using the Ribosomal Database Project database (RDP, version 10.27)^38^. Thresholds for the grouping of sequences at phylum and genus levels were set to intra-sequence identities of 76% and 97%, respectively^39^.

Protein-encoding regions (open reading frames (ORFs)) were predicted by using MetaGeneAnnotator software^40^. The predicted ORFs were annotated by BLASTP by using the Kyoto Encyclopedia of Genes and Genomes (KEGG) databases^41^ and an E value of ≤10^−5^.

Carbon-fixation and nitrogen-cycling gene sequences were chosen from the sequences with the assigned KEGG orthology identifier (K-number). These sequences were normalised by the average complete length of each gene by using data from the NCBI complete genome database, before the relative abundance with respect to the total number of predicted ORFs was calculated.

The members of carbon and nitrogen fixers using the *rbcL*, *porA* and *nifH* genes with assigned sequences were predicted with BLASTP using the NCBI non-redundant database.

### Statistical analysis

R, R^2^ and regression p values of the regression model were calculated by using StatPlus:mac LE (AnalystSoft Inc., CA, USA).

The relative abundance data of the microbial community structure and functional gene annotation for each sample were used in the calculation of the Bray–Curtis dissimilarity index by using R package vegan (http://cc.oulu.fi/~jarioksa/softhelp/vegan/html/vegdist.html). Dissimilarity indices of the microbial community structure were visualised as the distance of hierarchical clusters using by R package pvclust^42^. A box plot was generated to compare the functional gene dissimilarity indices among different hierarchal functional categories from the SEED-43, COG-44 and KEGG-annotated datasets via the MG-RAST server^45^ (http://metagenomics.anl.gov/?page=Home). The KEGG functional hierarchy was determined from the KEGG BRITE hierarchy (http://www.genome.jp/kegg/kegg3b.html).

## Additional Information

**Accession codes:** The pyrosequencing reads were deposited in the DDBJ Sequence Read Archive database under accession number DRA001199.

**How to cite this article**: Fujimura, R. *et al.* Unique pioneer microbial communities exposed to volcanic sulfur dioxide. *Sci. Rep.* **6**, 19687; doi: 10.1038/srep19687 (2016).

## Supplementary Material

Supplementary Information

Supplementary Table S4

Supplementary Table S5

## Figures and Tables

**Table 1:** Chemical properties of the volcanic ash deposits and a forested soil.

Sampling site	Deposit age (year)	Sample name	pH (H_2_O)	Total carbon content (mg g-dw^−1^)	Total nitrogen content (mg g-dw^−1^)	SO_4_^2−^ concentration (mg g-dw^−1^)	Total Fe^2+^ concentration (mg g-dw^−1^)
OY	3.5^*^	3.5-OYVD	3.5^*^	0.2^*^	<0.1^*^	45.0	0.63
	6.6^*^	6.6-OYVD	3.4^*^	<0.1^*^	<0.1^*^	50.0	0.42
	9.5	9.5-OYVD	3.9	0.5	0.1	47.5	0.33
IG	8.9	IGVD	4.3	1.6	0.2	40.0	0.31
CL	>800^*^	CLS	6.1^*^	41.3^*^	3.7^*^	0.38	0.15

*Data from Fujimura *et al*., 2012^9^
