# Supplementary material for: Unique pioneer microbial communities exposed to volcanic sulfur dioxide
Source: Sci Rep. 2016 Jan 21;6:19687. doi: 10.1038/srep19687 (PMC4726209; doi:10.1038/srep19687)
Supplement: Supplementary Information [file srep19687-s1.pdf]

Supplementary information

Unique pioneer microbial communities exposed to volcanic sulfur dioxide

Reiko Fujimura[1†], Seok-Won Kim[2††], Yoshinori Sato[3], Kenshiro Oshima[2], Masahira Hattori[2], Takashi Kamijo[4], Hiroyuki Ohta[1]

[1]Ibaraki University College of Agriculture, Ibaraki 300-0332, Japan. [2]Department of Computational Biology, Graduate School of Frontier Science, The University of Tokyo, Kashiwa, Chiba 277-8568, Japan. [3]National Research Institute for Cultural Properties, Tokyo, Tokyo, 110-8713, Japan. [4]Graduate School of Life and Environmental Science, University of Tsukuba, Tsukuba, Ibaraki 305-8572, Japan.

Present address
[†]Atmosphere and Ocean Research Institute, University of Tokyo, Kashiwa, Chiba 277-8564, Japan.
[††]Laboratory for Integrated Bioinformatics, RIKEN Center for Integrative Medical Sciences, Yokohama, Kanagawa, 230-0045, Japan

Table S1 | Monthly average volcanic SO_2 gas emission of every years since 2004 in Miyake-jima.

Observational year	Average emission of SO2 gas*1 (tons month^{-1})
2004	5,652
2005	4,086
2006	2,353
2007	2,295
2008	1,811
2009	1,407
2010	1,024
2011	826
2012	733
2013	431
2014	273

*1Original data from Japan Meteorological Agency (http://www.data.jma.go.jp/svd/vois/data/tokyo/320_Miyakejima/320_So2emission.htm) .

Table S2 | Numbers and percentages of SO_2 gas detection count at each concentration for each site during the monitoring period.

Site	OY	IG1	CL
Total number of recording count	1,521	1,518	2,821
Total number of SO_2 detection count	272	18	0
Percentage of SO_2 detection count of each concentration*			
Detection range (ppm)		%	
≥ 1 ppm	55.9	19.5	0.0
≥ 2 ppm	29.4	1.1	0.0
≥ 3 ppm	11.4	0.0	0.0
≥ 4 ppm	2.9	0.0	0.0
≥ 5 ppm	0.4	0.0	0.0

*Percentages were calculated from the SO_2 detection counts at each concentration divided by the total number of SO_2 detection counts.

Table S3 | Summary of pyrosequence dataset.

Sample	Number of fragments	Average length of fragments	mean GC %	No of predicted ORFs	Average length of amino acid residues	Number of 16S rRNA gene fragments	% of KEGG assigned
3.5-OYVD	598,685	393	55 ± 10	696,999	83	464	28.9
6.6-OYVD	253,003	373	59 ± 8	292,431	83	396	31.1
9.5-OYVD	1,213,490	402	57 ± 10	1,350,554	87	1,078	26.1
IGVD	902,121	380	59 ± 10	1,024,620	83	463	23.6
CLS	874,208	439	62 ± 7	1,057,070	97	296	30.4

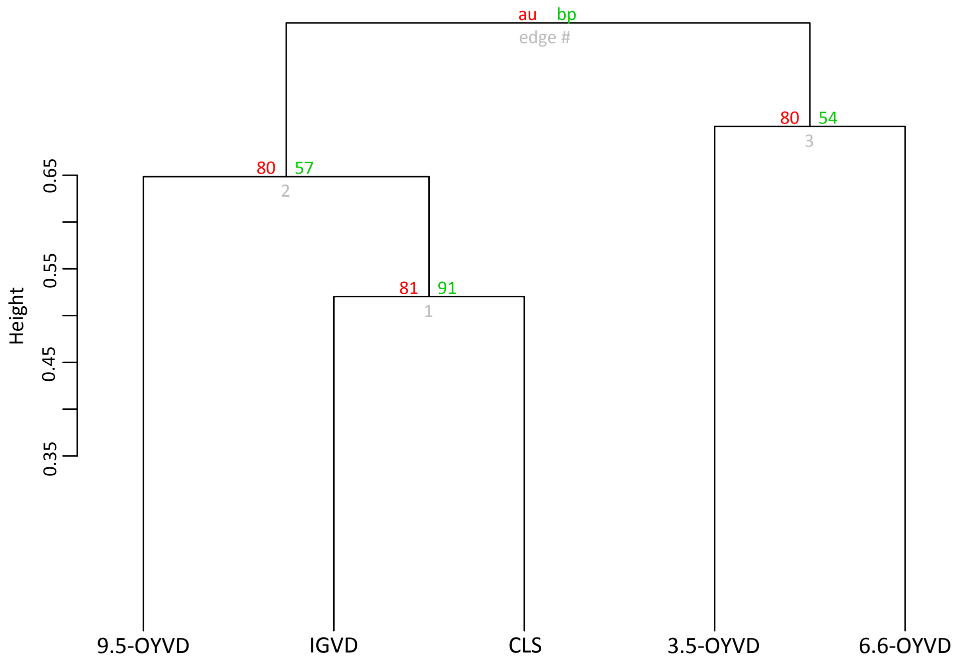

Figure S1 | Hierarchical cluster analysis using Bray−Curtis dissimilarity of the taxonomic abundance data of 16S rDNA. Numbers on branches indicate p-values of Approximately Unbiased and Bootstrap Probability (green). Definitions are detailed in Supplementary Information; also see pvclust site (http://www.sigmath.es.osaka-u.ac.jp/shimo-lab/prog/pvclust/).

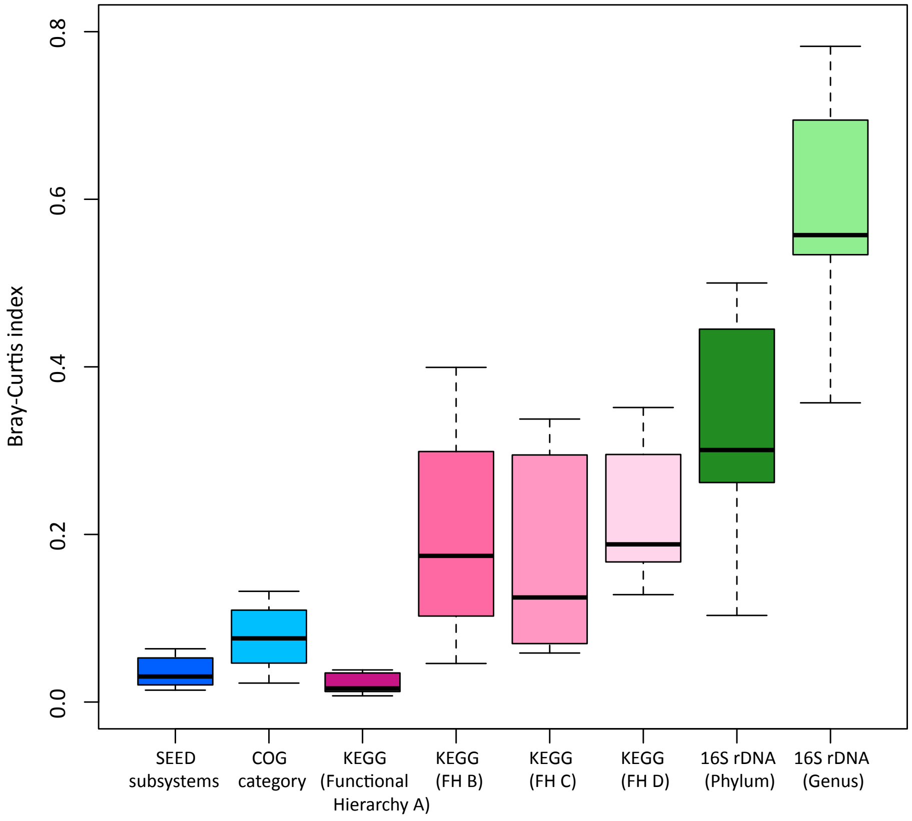

Figure S2 | Box plot of Bray−Curtis dissimilarity indices. Each box plot indicates the variability of Bray −Curtis indices of the annotated gene relative abundance in each functional category. Analyzed database names are shown on the X axis. The indices were calculated from the relative abundance of functional gene categories. Metagenomic reads were functionally annotated using the functional category databases of the SEED subsystems[43], the Clusters of Orthologous Groups (COG) functional categories[44], and the Kyoto Encyclopedia of Gene and Genomes (KEGG) classification[41] with the metagenomics RAST server (MG-RAST; https://metagenomics.anl.gov/)[45]. The latter assigns four levels of functional hierarchy (FH) based on the KEGG BRITE hierarchy (http://www.genome.jp/kegg/kegg3b.html): “A” is the largest category of biochemical reactions such as “metabolism” and “genetic information processing” as the highest hierarchy; “B” is the larger category of metabolic pathways such as “carbohydrate metabolism” and “amino acid metabolism;” “C” is the smaller category of metabolic pathways such as the “TCA cycle” and “Lysine biosynthesis;” “D” is the functional units (i.e., each gene, the smallest category) such as “*rbcL*” and “*nifH*” as the lowest hierarchy.
